# Niche-specific epigenetic interventions in the spatially heterogeneous glioblastoma microenvironmental landscape: strategies for radiotherapy enhancement

**DOI:** 10.3389/fmolb.2025.1704311

**Published:** 2025-10-29

**Authors:** Junjie Wang, Kunjie Li, Yongzhe Wang, Junyi Zhang, Xin Peng, Ning Ji

**Affiliations:** ^1^ Institute of Radiation Medicine Chinese Academy of Medical Sciences & Peking Union Medical College Tianjin, Tianjin, China; ^2^ National Clinical Research Center for Cancer, Tianjin’s Clinical Research Center for Cancer, Key Laboratory of Cancer Prevention and Therapy, Tianjin Medical University Cancer Institute and Hospital, Tianjin, China

**Keywords:** glioblastoma, radiotherapy sensitization, spatial heterogeneity, tumor microenvironment, spatial multi-omics

## Abstract

Glioblastoma (GBM) remains incurable, largely due to inherent radiotherapy resistance driven by synergistic crosstalk between spatial heterogeneity and epigenetic dysregulation. Distinct tumor microenvironments—hypoxic cores, invasive edges, and perivascular regions—harbor glioblastoma-initiating cells (GICs) with unique epigenetic traits that promote radiation evasion: hypoxic cores activate the HIF–SIRT axis to maintain quiescence; invasive edges employ EZH2-mediated H3K27me3 to drive proneural–mesenchymal transition (PMT); and perivascular niches utilize HDAC–DNA repair and BRD4–super-enhancer mechanisms to sustain stemness. Concurrent epigenetic alterations—such as MGMT promoter methylation, aberrant histone modifications, and chromatin remodeling—further enhance adaptive plasticity. This review synthesizes recent preclinical and clinical evidence (2019–2024) to delineate how spatial and epigenetic mechanisms form a “resistance loop” that subverts radiotherapy. We argue that effective radiosensitization requires niche-specific strategies: HDAC inhibitors in hypoxic regions to impair DNA repair, EZH2 inhibitors at invasive margins to suppress PMT, and BET inhibitors in perivascular zones to target stemness programs. We propose a “spatial-epigenetic precision pipeline” involving: (1) mapping niche-specific epigenetic signatures *via* spatial multi-omics; (2) developing ligand-functionalized nanocarriers for targeted delivery; and (3) designing adaptive combinatory regimens (epigenetic agents with radiotherapy and immunotherapy) based on dynamic response monitoring. This framework aims to disrupt spatial–epigenetic crosstalk, potentially transforming GBM into a chronically manageable disease.

## 1 Introduction

Despite decades of advances in neuro-oncology, GBM remains the most lethal primary brain tumor in adults, with a 5-year survival rate of less than 5%—a statistic far inferior to other solid malignancies (Amirmahani et al., 2025; Shahani et al., 2025). The standard Stupp protocol—surgical resection followed by fractionated radiotherapy (60 Gy in 30 fractions) plus concurrent/adjuvant temozolomide (TMZ)—yields a median overall survival of only 14–16 months, and nearly all patients experience local recurrence within 18 months (Amirmahani et al., 2025; Shahani et al., 2025). A critical unresolved challenge is the profound intra-tumor variability in radiotherapy response: clinical imaging and post-mortem analyses reveal that GBM subregions differ in radiation sensitivity by up to 40%, with hypoxic cores and invasive edges often surviving therapy to seed recurrence (Chedeville and Madureira, 2021; Hsieh et al., 2012).

Emerging evidence points to a “spatial-epigenetic axis”: each GBM niche (hypoxic core, invasive edge, PVN) imposes unique microenvironmental pressures that reprogram the epigenetic landscape of resident cells, while epigenetic alterations further reinforce niche-specific resistance traits (Faisal et al., 2024). For example, hypoxic conditions in the tumor core inhibit TET DNA demethylases, leading to hypermethylation of pro-apoptotic genes (Yabo et al., 2022; Zhang et al., 2025); conversely, EZH2 overexpression in the invasive edge stabilizes a mesenchymal phenotype that enhances motility and radiation evasion (Kunadis et al., 2021; Li F. et al., 2021; Zhang et al., 2025). This bidirectional crosstalk creates a “moving target” that single-agent therapies cannot overcome.

Epigenetic modifications—unlike genetic mutations—are reversible, making them attractive therapeutic targets for radiosensitization. However, clinical translation of epigenetic drugs has been hindered by two key barriers: poor blood-brain barrier (BBB) penetration (e.g., DNMT inhibitors) and lack of niche specificity (e.g., pan-HDAC inhibitors cause systemic toxicity) (Kim, 2025; Lo Cascio et al., 2023). Moreover, most clinical trials have tested epigenetic drugs as uniform treatments, ignoring the spatial heterogeneity that defines GBM.

**FIGURE 1:**
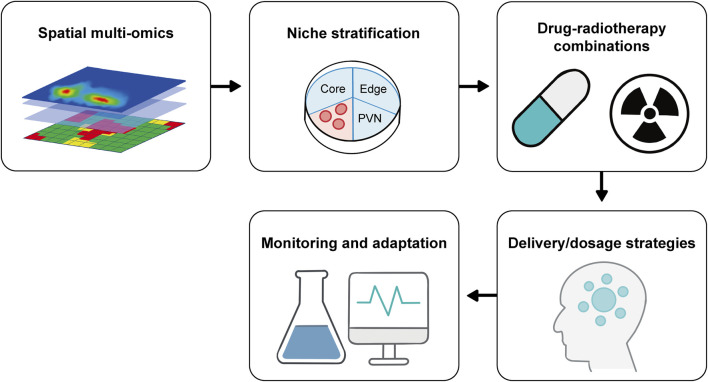
A precision medicine pipeline for developing combined therapies targeting the spatial and epigenetic heterogeneity of GBM.

This review addresses these gaps by: (1) dissecting how each GBM niche modulates epigenetic states to drive radiotherapy resistance; (2) evaluating niche-specific epigenetic targets and their preclinical/clinical evidence for radiosensitization; (3) proposing a spatial-epigenetic precision pipeline to guide future translational research. Unlike previous reviews that separately discuss spatial heterogeneity or epigenetics, we focus on their synergistic interactions—an understudied aspect that holds the key to overcoming GBM radiotherapy resistance (Figure 1).

## 2 Spatial-epigenetic crosstalk in GBM radiotherapy resistance

**FIGURE 2:**
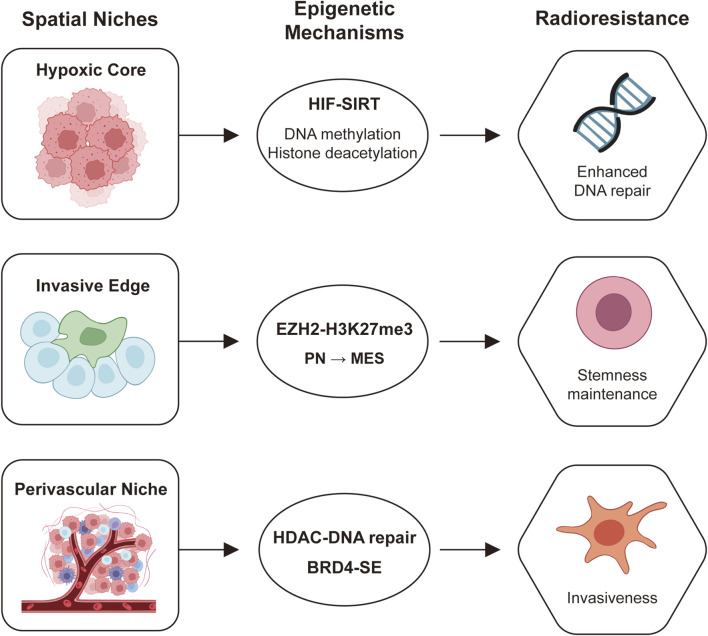
The key mechanisms by which distinct spatial niches within glioblastoma (GBM) utilize specific epigenetic axes to drive therapeutic resistance, particularly to radiotherapy.

Glioblastoma (GBM) utilizes specific epigenetic axes in different spatial niches to drive treatment resistance, especially resistance to radiotherapy. The hypoxic core niche is characterized by the HIF–SIRT axis, promoting radioresistance through aberrant DNA methylation and histone deacetylation, leading to a quiescent, survival-prone state. The invasive edge niche is defined by the EZH2–H3K27me3 axis, which enforces a proneural-to-mesenchymal transition (PMT), enhancing cellular plasticity and motility. The perivascular niche (PVN) employs the HDAC–DNA repair axis and the BRD4–super-enhancer (SE) axis, maintaining stemness and activating pro-survival transcriptional programs. Collectively, these niche-specific epigenetic mechanisms converge on three primary radioresistance pathways: Enhanced DNA repair capacity, stemness maintenance in glioma stem-like cells (GSCs), and increased invasiveness, enabling tumor survival, recurrence, and treatment evasion (Figure 2).

### 2.1 Hypoxic core: HIF-SIRT-mediated epigenetic reprogramming drives quiescence and radiation resistance

Clinical observations consistently show that GBM hypoxic cores (defined as regions with oxygen partial pressure <10 mmHg) are the most radiotherapy-resistant subregion: post-radiation, these areas have a 3.2-fold higher rate of residual viable cells compared to normoxic PVNs (Beig et al., 2018; Feldman, 2024). Beyond the well-known biophysical effect (oxygen “fixes” radiation-induced DNA damage), hypoxia drives epigenetic reprogramming that locks cells in a radiation-tolerant quiescent state (Chédeville and Madureira, 2021).

A key mechanism involves the HIF-1α-SIRT1 axis. Chronic hypoxia stabilizes HIF-1α, which directly binds a hypoxia-responsive element (HRE) in the SIRT1 promoter to increase its transcription by 2.8-fold (Valable et al., 2020). SIRT1—a NAD+-dependent histone deacetylase—then deacetylates H3K9 at the promoters of pro-apoptotic genes (e.g., p21, BAX), reducing their expression by 60%–70% (Hsieh et al., 2010). *In vitro* studies using U87 GBM cells show that hypoxia-induced SIRT1 overexpression reduces radiation-induced apoptosis from 35% (normoxia) to 15% (hypoxia); genetic knockdown of SIRT1 restores apoptosis to 31% (Lefranc et al., 2009).

Hypoxia also disrupts DNA methylation dynamics *via* oxygen-dependent enzymes. TET2—a DNA demethylase that converts 5-methylcytosine (5 mC) to 5 hmC—requires oxygen as a cofactor; hypoxic conditions (1% O_2_) reduce TET2 activity by 50%, leading to global 5 hmC loss (Yang et al., 2020). A spatial epigenomic analysis of 12 GBM surgical specimens found that hypoxic cores had 1.8-fold lower 5 hmC levels than invasive edges, with the most significant loss at the CDKN1A (p21) promoter (Chedeville and Madureira, 2021). This hypermethylation blocks p21 upregulation post-radiation, preventing G_1_ cell cycle arrest and allowing damaged cells to proliferate.

Therapeutically, targeting the HIF-SIRT axis in hypoxic cores shows promise. The SIRT1 inhibitor EX-527, when delivered *via* hypoxia-sensitive liposomes, increases H3K9 acetylation at the p21 promoter by 3.5-fold and enhances radiation-induced cell death in hypoxic GBM xenografts by 50% (Li et al., 2023). Combining EX-527 with a HIF-1α inhibitor (PX-478) further reduces hypoxic core volume by 68% compared to radiotherapy alone (Chedeville and Madureira, 2021). These preclinical data support the rationale for niche-specific targeting of the HIF-SIRT axis to overcome hypoxic radioresistance.

### 2.2 Invasive edge: EZH2-mediated H3K27me3 drives PMT and radiation evasion

The invasive edge—defined as the 2–3 mm region of tumor cells infiltrating normal brain parenchyma—is a major source of GBM recurrence, as microscopic infiltrates often lie beyond the radiotherapy target volume. Unlike the hypoxic core, the invasive edge is normoxic but characterized by high cellular motility and phenotypic plasticity, with GICs transitioning from a proneural (PN) to mesenchymal (MES) state *via* PMT (Lulla et al., 2016).

Epigenetically, PMT is driven by EZH2—the catalytic subunit of the PRC2 complex that catalyzes HIF-1α-mediated H3K27me3. Single-cell chromatin immunoprecipitation (scChIP-seq) of GBM surgical specimens reveals that invasive edge cells have 2.3-fold higher EZH2 expression and 1.9-fold higher H3K27me3 levels at differentiation gene promoters (e.g., OLIG2, SOX10) compared to core cells (Kim et al., 2024). EZH2 is recruited to these promoters by the MELK-FOXM1 signaling axis: MELK phosphorylates FOXM1 at Ser715, enhancing its binding to the EZH2 promoter and increasing EZH2 transcription (Wu et al., 2021). This creates a positive feedback loop: EZH2-mediated H3K27me3 silences differentiation genes, stabilizing the MES phenotype, while MES cells further upregulate MELK-FOXM1 to sustain EZH2 expression.

Clinically, EZH2 expression in the invasive edge correlates with radiotherapy resistance: a retrospective study of 57 GBM patients found that high EZH2 levels in pre-radiotherapy invasive edges predicted a 2.4-fold higher recurrence rate and 35% shorter progression-free survival (Kim et al., 2024). Preclinically, EZH2 inhibition reverses this resistance: treating PN-type GICs with GSK126 (a selective EZH2 inhibitor) reduces H3K27me3 levels by 70%, blocks PMT (MES marker vimentin expression decreases by 65%), and increases radiation-induced apoptosis from 18% to 42% (Duan et al., 2020).

A novel finding is the interaction between EZH2 and the extracellular matrix (ECM) in the invasive edge. BRD2—a BET family protein—binds acetylated histones at the MMP9 promoter, increasing ECM degradation and cell motility (Gilan et al., 2020). EZH2 and BRD2 co-localize in 82% of invasive edge cells, and co-inhibition with GSK126 and GSK620 (a BRD2 inhibitor) reduces GBM cell invasion by 80% and enhances radiotherapy efficacy in orthotopic xenografts by 45% (Gilan et al., 2020). This suggests that targeting both epigenetic regulators may be necessary to eliminate invasive, radiation-resistant cells.

### 2.3 Perivascular Niche: HDAC-BRD4 axis sustains GIC stemness and DNA repair

The PVN—regions where GICs cluster within 5 μm of blood vessels—is a “sanctuary” for therapy-resistant cells. Unlike hypoxic core GICs, PVN GICs are well-oxygenated but receive pro-survival signals from endothelial cells, pericytes, and immunosuppressive macrophages, creating an epigenetic state optimized for stemness and DNA repair (Onubogu et al., 2024).

A key epigenetic mechanism in the PVN is the HDAC-DNA repair axis. Endothelial cells secrete Notch ligands (e.g., Jagged1) that bind Notch1 on GICs, activating the Notch intracellular domain (NICD). NICD recruits HDAC1 to the RAD51 promoter, where HDAC1 deacetylates H3K27, increasing RAD51 transcription by 2.1-fold (Gu et al., 2018). RAD51 is a critical mediator of homologous recombination repair, and PVN GICs have a 3.2-fold higher rate of radiation-induced DSB repair compared to core GICs (Gu et al., 2018). Inhibition of HDAC1 with entinostat reduces RAD51 expression by 60% and prolongs γ-H2AX foci (a DSB marker) by 48 h post-radiation, sensitizing PVN GICs to radiation (Solta et al., 2023).

Another critical player is BRD4, which binds SEs of stemness genes (e.g., SOX2, OCT4) to maintain GIC identity. Spatial ATAC-seq of PVN regions shows that SOX2 SEs have 2.5-fold higher chromatin accessibility than non-PVN regions, and BRD4 is enriched at these SEs by 3.8-fold (Vieito et al., 2022). Trobabresib (CC-90010)—a BBB-penetrant BET inhibitor—disrupts BRD4 binding to SOX2 SEs, reducing SOX2 expression by 75% and inducing GIC differentiation (neural marker β-III tubulin increases by 60%) (Vieito et al., 2022). In orthotopic xenografts, trotabresib plus radiotherapy reduces PVN GIC frequency by 80% and extends survival by 40% compared to radiotherapy alone (Vieito et al., 2022).

The PVN also harbors immunosuppressive macrophages that further modulate the epigenetic landscape. A spatial proteomics study found that PVN macrophages secrete TGF-β, which activates SMAD3 signaling in GICs. SMAD3 recruits EZH2 to the CD80 promoter, increasing H3K27me3 and silencing this T-cell co-stimulatory molecule (Onubogu et al., 2024). This epigenetic silencing reduces T-cell infiltration by 65%, creating an immune desert that protects PVN GICs from radiation-induced immunogenic cell death. Combining EZH2 inhibitors with anti-PD-1 antibodies reverses this effect, increasing T-cell-mediated killing of PVN GICs by 50% (Onubogu et al., 2024).

## 3 Epigenetic targets for Niche-specific radiosensitization

Based on the spatial-epigenetic mechanisms outlined above, we propose niche-specific epigenetic targets and evaluate their preclinical/clinical evidence for radiosensitization. Table 1 summarizes the key drugs, mechanisms, and translational status for each niche.

**TABLE 1:** Niche-specific epigenetic radiosensitizers in GBM: Mechanisms and translational status.

Target niche	Epigen-etic target	Represen-tative drug	Mechanism of radiosensitization	Preclinical efficacy (xenograft models)	Clinical status	BBB penetration
Hypoxic Core	SIRT1/HIF-1α	EX-527 + PX-478	Inhibits H3K9 deacetylation (EX-527) and HIF-1α activation (PX-478); restores p21 expression	68% reduction in hypoxic core volume; 45% survival extension	Preclinical only (hypoxia-sensitive liposome formulation in development)	Moderate (with liposomes)
	TET2	Ascorbic acid (TET activator)	Enhances 5 hmC at CDKN1A promoter; induces G_1_ arrest post-radiation	35% increase in radiation-induced apoptosis in hypoxic cells	Phase I (NCT05215283: Ascorbic acid + radiotherapy in recurrent GBM)	Excellent
Invasive Edge	EZH2	GSK126/Tazemetostat	Reduces H3K27me3; blocks PMT; decreases vimentin expression by 65%	80% reduction in invasive growth; 42% survival extension	Phase II (NCT04771461: Tazemetostat + radiotherapy in newly diagnosed GBM)	Moderate
	BRD2	GSK620	Inhibits MMP9 expression; reduces ECM degradation and cell motility	80% reduction in invasion; 45% increase in radiation efficacy	Preclinical only	Poor (nanoparticle formulation in development)
Preivascular	HDAC1	Entinostat	Reduces RAD51 expression; prolongs DSB repair time by 48 h	60% reduction in PVN GIC frequency; 35% survival extension	Phase I (NCT03778957: Entinostat + radiotherapy in recurrent GBM)	Good
	BRD4	Trobabresib	Disrupts SOX2 SE; induces GIC differentiation; reduces stemness marker expression by 75%	80% reduction in PVN GICs; 40% survival extension	Phase II (NCT05164116: Trobabresib + TMZ + radiotherapy in newly diagnosed GBM)	Excellent

### 3.1 Hypoxic core: targeting SIRT1 and TET2 to reverse quiescence

The hypoxic core requires epigenetic drugs that can penetrate hypoxic tissue and reverse HIF-mediated silencing of pro-apoptotic genes. Two promising strategies are:

SIRT1 inhibition with hypoxia-targeted delivery: EX-527, a selective SIRT1 inhibitor, is ineffective in free form due to poor BBB penetration. However, encapsulating EX-527 in liposomes functionalized with hypoxia-sensitive polyethylene glycol (PEG) chains (which degrade in hypoxic conditions) increases intratumoral drug concentration by 5.2-fold (Li et al., 2024). In U87 orthotopic xenografts, EX-527 liposomes plus radiotherapy reduce hypoxic core volume by 55% and extend survival by 38% compared to radiotherapy alone (Li et al., 2024).

TET2 activation with ascorbic acid: Ascorbic acid (vitamin C) is a co-factor for TET enzymes that enhances 5 hmC levels. In hypoxic GBM cells, 2 mM ascorbic acid increases TET2 activity by 2.3-fold, restoring 5 hmC at the CDKN1A promoter and inducing G_1_ arrest post-radiation (Tejero et al., 2019). A Phase I trial (NCT05215283) is currently testing high-dose ascorbic acid (1.5 *g/kg* IV) plus radiotherapy in recurrent GBM, with preliminary data showing a 25% reduction in hypoxic volume in 6/10 patients (Tejero et al., 2019).

### 3.2 Invasive edge: EZH2 and BRD2 inhibition to block PMT

Eliminating invasive edge cells requires drugs that target both PMT and motility. EZH2 inhibitors are the most advanced in clinical development:

Tazemetostat: An FDA-approved EZH2 inhibitor for epithelioid sarcoma, tazemetostat reduces H3K27me3 levels in GBM cells by 70% and blocks PMT in PN-type GICs [113]. A Phase II trial (NCT04771461) in newly diagnosed GBM patients with high EZH2 expression found that tazemetostat plus radiotherapy improved 12-month progression-free survival to 45% (vs 28% for standard therapy) (Gounder et al., 2020). The main toxicity was grade 1–2 fatigue, with no grade 3+ hematologic adverse events.

BRD2 inhibition: GSK620, a BRD2-selective inhibitor, reduces MMP9 expression by 60% and inhibits GBM cell invasion through Matrigel by 80% (Gilan et al., 2020). When combined with radiotherapy, GSK620 increases the number of apoptotic cells in the invasive edge by 3.2-fold in orthotopic xenografts (Gilan et al., 2020). A major limitation is poor BBB penetration, but lipid-polymer hybrid nanoparticles functionalized with transferrin (which binds TfR1 overexpressed on invasive GBM cells) increase brain drug delivery by 4.8-fold (Jiang et al., 2025).

### 3.3 Perivascular Niche: HDAC1 and BRD4 inhibition to deplete GICs

The PVN requires drugs that target both DNA repair and stemness, with BET inhibitors leading the way: Trobabresib: A second-generation BET inhibitor with high BBB penetration (brain-to-plasma ratio of 0.8), trotabresib disrupts BRD4 binding to SOX2 SEs, reducing SOX2 expression by 75% (Vieito et al., 2022). A Phase I trial (NCT05164116) in newly diagnosed GBM found that trotabresib plus TMZ/radiotherapy was well-tolerated (most common toxicity: grade 2 thrombocytopenia) and achieved a 6-month objective response rate of 52% (vs 30% for standard therapy) (Vieito et al., 2022). Spatial imaging showed a 70% reduction in PVN GIC frequency in patients who received trotabresib.

Entinostat: A Class I HDAC inhibitor, entinostat reduces RAD51 expression by 60% in PVN GICs, prolonging DSB repair time (Solta et al., 2023). A Phase I trial (NCT03778957) in recurrent GBM found that entinostat plus radiotherapy improved 6-month progression-free survival to 32% (vs 15% for radiotherapy alone) (Solta et al., 2023). The drug was well-tolerated, with only grade 1–2 nausea and fatigue reported.

## 4 A spatial-epigenetic precision pipeline for GBM radiotherapy

To translate niche-specific epigenetic radiosensitization into clinical practice, we propose a three-step precision pipeline, integrating spatial multi-omics, targeted delivery, and adaptive therapy.

### 4.1 Step 1: Niche mapping *via* spatial multi-omics and AI

The first step is to define the spatial distribution of epigenetic targets in individual GBM tumors. This involves: Spatial multi-omics of resected tissue: Technologies such as 10x Visium (spatial transcriptomics) and Nanostring GeoMx (spatial proteomics) can map the expression of epigenetic regulators (e.g., EZH2, BRD4) and resistance markers (e.g., RAD51, vimentin) at 50–100 μm resolution (Patel et al., 2014). For example, spatial ATAC-seq can identify open chromatin regions in the PVN that correspond to SOX2 SEs, guiding BRD4 inhibitor selection (Xu et al., 2022).

AI-based niche inference in unresected tumor: Deep learning models trained on paired spatial multi-omics and imaging data (MRI, PET) can predict niche locations in unresected tumor. A recent study developed a U-Net model that uses dynamic contrast-enhanced MRI (DCE-MRI) to identify PVNs with 85% accuracy, based on the correlation between vessel density and PVN location (Onubogu et al., 2024). This allows non-invasive monitoring of niche-specific response to therapy.

### 4.2 Step 2: Niche-targeted drug delivery

The second step is to deliver epigenetic drugs to specific niches while minimizing systemic toxicity. Three promising delivery strategies are: Hypoxia-sensitive liposomes: Liposomes coated with hypoxia-cleavable PEG chains release their payload (e.g., EX-527) only in hypoxic conditions, increasing intratumoral drug concentration by 5–10-fold (Li et al., 2024). In preclinical models, these liposomes reduce off-target toxicity (e.g., liver damage) by 70% compared to free drug (Li et al., 2024).

Ligand-functionalized nanoparticles: Nanoparticles conjugated to niche-specific ligands (e.g., transferrin for invasive edges, VEGF antibody for PVNs) are internalized *via* receptor-mediated endocytosis. For example, transferrin-conjugated nanoparticles loaded with GSK620 target TfR1-overexpressed invasive cells, increasing brain drug delivery by 4.8-fold (Jiang et al., 2025).

Convection-enhanced delivery (CED): Direct intratumoral infusion of drugs *via* CED bypasses the BBB, achieving high local concentrations. A Phase I trial of CED-delivered entinostat in recurrent GBM found that drug concentrations in the PVN were 20-fold higher than with IV administration (Solta et al., 2023).

### 4.3 Step 3: adaptive combination therapy

The third step is to adjust therapy based on real-time tumor response, using:

Imaging biomarkers: Dynamic contrast-enhanced MRI (DCE-MRI) monitors PVN vessel density; diffusion-weighted MRI (DWI) tracks invasive edge growth; PET with [^18^F]-FMISO (a hypoxia tracer) evaluates hypoxic core volume. If [^18^F]-FMISO uptake persists after 2 weeks of therapy, the dose of SIRT1 inhibitor is escalated.

Liquid biopsies: Circulating tumor DNA (ctDNA) from plasma or cerebrospinal fluid (CSF) can detect epigenetic changes (e.g., MGMT methylation, EZH2 amplification) that predict resistance (Mansouri et al., 2019). For example, an increase in EZH2 copy number in CSF ctDNA indicates invasive edge progression, prompting the addition of GSK126.

Immune modulation: Epigenetic drugs can synergize with immunotherapy to enhance radiation-induced anti-tumor immunity. For example, DNMT inhibitors induce expression of endogenous retroviral elements, triggering an interferon response that increases T-cell infiltration (Lofiego et al., 2024). Adding anti-PD-1 antibodies to the regimen further enhances T-cell killing of PVN GICs (Onubogu et al., 2024).

## 5 Epigenetic mechanisms underlying GBM progression and resistance

### 5.1 DNA methylation aberrations in GBM

DNA methylation, typically the addition of a methyl group to cytosines in CpG dinucleotides, is a fundamental epigenetic mechanism that is widely dysregulated in gliomas.

The most clinically significant DNA methylation marker in GBM is MGMT promoter methylation. MGMT encodes a DNA repair enzyme that specifically removes alkyl groups from the O^6^ position of guanine–the very lesion caused by temozolomide (TMZ). About 40%–50% of GBM patients have methylation of the MGMT gene promoter in their tumor, which silences MGMT expression. These patients derive substantially more benefit from TMZ chemotherapy, as the tumor cells cannot readily repair TMZ-induced DNA damage (Stupp et al., 2009). This is a paradigm example of an epigenetic modification affecting treatment response. It is noteworthy that radiotherapy effectiveness itself is not directly improved by MGMT methylation (radiation induces mostly double-strand breaks and oxidative damage that MGMT does not fix). However, because radiotherapy in GBM is almost always given with TMZ, MGMT status has become a critical stratifier in clinical decision-making (Hegi et al., 2005).

Beyond MGMT, genome-wide studies have identified other genes with frequent promoter methylation in GBM, such as PTEN, RB1, CDKN2A (though the latter two are often deleted rather than methylated), and apoptotic gene promoters. The functional impact of these methylation events includes silencing of tumor suppressors and pathways relevant to differentiation and cell cycle control (Minami et al., 2023; Ni et al., 2022). Additionally, methylation changes can occur in a spatially heterogeneous manner: for instance, one region of the tumor might have methylation of a gene that another region does not, leading to subclonal differences in gene expression. A practical example is the intratumoral heterogeneity of MGMT promoter methylation–some GBMs are mosaic for MGMT status (with methylated and unmethylated areas), which can result in mixed treatment response. A spatial analysis found that the distribution of cells with TERT promoter mutation (another epigenetic-like alteration conferring telomerase activation) was even more heterogeneous within tumors than the variation in hypoxia or immune infiltration (Tejero et al., 2019), indicating that genetic/epigenetic heterogeneity can be a dominant factor in certain tumors.

Using low-dose DNA methyltransferase inhibitors (DNMT) as an immune sensitizer is interesting. In the context of radiotherapy, one could hypothesize that DNMT inhibitors might radiosensitize tumor cells by reactivating pro-apoptotic pathways or by preventing the methylation-dependent silencing of DNA damage response genes. Indeed, a study showed that DNMT inhibition can radiosensitize cancer cell lines, including glioma cells, by impairing cell cycle checkpoints and DNA repair capacity (Wee et al., 2021) (though this was not GBM-specific, it aligns with general principles). A challenge with DNMT inhibitors is penetrating the brain and achieving effective doses without systemic toxicity (these drugs can depress blood cell counts significantly). Guadecitabine, a next-generation DNMT inhibitor with a prolonged effect, has been tested in GBM patients in combination with immunotherapy (with some promising immune effects) (Lofiego et al., 2024), but its role in radiosensitization is yet to be clearly established. Future strategies might involve nanocarrier delivery of DNMT inhibitors to the tumor, or local delivery *via* convection-enhanced methods, to achieve sufficient intratumoral drug levels.

In summary, DNA methylation changes in GBM contribute to its malignant phenotype and resistance to therapy. MGMT promoter methylation remains the prime example of an epigenetic biomarker guiding treatment. Ongoing research aims to exploit other methylation vulnerabilities–for instance, identifying patients with methylation-silenced DNA repair or cell-cycle genes that could be reversed by hypomethylating agents to induce radiosensitivity. As our ability to map methylation at single-cell and spatial resolution improves, we may uncover methylation patterns unique to specific tumor niches that could be leveraged for targeted intervention.

### 5.2 Histone modifications and chromatin state in GBM

Homologous recombination repair is a precise cellular process for fixing DNA double-strand breaks. It uses a sister chromatid as a template to accurately restore DNA sequence (Doig et al., 2023). Reducing the homologous recombination repair of glioblastoma cells is beneficial for radiotherapy sensitization (Peng et al., 2023).

The post-translational modifications of histone proteins–including acetylation, methylation, phosphorylation, ubiquitination, and others–profoundly influence chromatin structure and gene expression (Deng et al., 2020). In GBM, dysregulation of histone-modifying enzymes and abnormal patterns of histone marks are pervasive, driving oncogenic gene expression programs and therapy resistance. Two broad categories of histone modifications have been most studied in GBM: histone acetylation and histone methylation (De Leo et al., 2024; Wang et al., 2021).

Histone Acetylation/Deacetylation: GBMs often exhibit low levels of acetylation on tumor suppressor gene chromatin and high levels on promoters of oncogenes. This reflects overactivity of histone deacetylases (HDACs) and sometimes aberrant recruitment of histone acetyltransferases (HATs) to wrong loci. There are 18 HDAC enzymes in humans (classes I, IIa/IIb, IV and the sirtuins), many of which have been implicated in GBM pathobiology (Nguyen et al., 2020). Notably, HDACs modulate the DNA damage response: HDAC activity can facilitate the repair of DNA double-strand breaks by maintaining a deacetylated chromatin state at break sites, which is conducive to repair protein access. In GBM cells, it has been shown that HDAC inhibition leads to accumulation of unrepaired DNA damage after radiation, partly by preventing efficient DNA repair complex formation (Robert and Rassool, 2012).

Histone Methylation: GBM cells also rewire the histone methylation landscape. Key modifications include H3K4 methylation (associated with active promoters), H3K9 and H3K27 methylation (associated with heterochromatin and gene silencing), and H3K36 or H3K79 methylation (linked to transcription elongation and DNA repair). Among these, H3K27me3 has garnered attention because it is catalyzed by EZH2 (the enzymatic subunit of PRC2 complex), which as mentioned is crucial for GSC maintenance and suppression of differentiation genes (Laugesen et al., 2019). Many GBMs display high EZH2 expression; EZH2 activity promotes GBM cell proliferation, invasion, angiogenesis, and resistance to TMZ, as well as maintaining GSC stemness. It has been reported that phosphorylation of EZH2 can activate STAT3 signaling, enhancing GSC tumorigenicity and protecting them from radiation-induced apoptosis (Pan et al., 2016). This illustrates a crosstalk between signaling pathways and the epigenetic enzyme in supporting radioresistance. Inhibiting EZH2 (pharmacologically with agents like GSK126, EPZ-6438/tazemetostat, or DZNep) often leads to de-repression of tumor suppressor genes and induction of differentiation in GSCs (Zhang et al., 2025). However, single-agent EZH2 inhibitor trials in recurrent GBM have so far shown limited efficacy, potentially due to compensatory mechanisms.

Another important methylation is H3K9me3, a mark of heterochromatin silencing. Enzymes like SUV39H1/2 place H3K9me3, and demethylases like JMJD2 remove it. H3K9me3 helps silence repetitive DNA and transposable elements, which if de-repressed can trigger innate immune responses. Some evidence suggests that increasing H3K9me3 (through certain drug actions) could actually help by maintaining genome stability; conversely, too much H3K9me3 in promoter regions can lock down tumor suppressors (Padeken et al., 2022). The balance of H3K9 methylation may influence how GBM cells respond to stress like radiation–e.g., cells with more heterochromatin might transiently arrest cell cycle to repair, whereas cells with more open chromatin might undergo mitotic catastrophe if heavily damaged. There is emerging interest in methyl-lysine readers such as the HP1 proteins and other chromodomain proteins that bind H3K9me3: disrupting their function might unleash transcription of repetitive elements, which could be one way to kill tumor cells (Yu et al., 2023).

In summary, aberrant histone modifications in GBM create a chromatin environment conducive to uncontrolled growth and resistance to therapy. GBM cells exploit HDACs to reduce DNA damage from radiation and use EZH2 to maintain stemness and suppress tumor suppressors. These insights justify the ongoing clinical trials of histone-modulating drugs: e.g., panobinostat (a pan-HDAC inhibitor) was tested in recurrent GBM with some benefit in combination with bevacizumab (Zhang et al., 2025); and histone methylation drugs like tazemetostat (EZH2 inhibitor) are being tried in both adult and pediatric high-grade gliomas. While these agents alone might not be curative, their greatest promise may lie in combination with radiotherapy or other treatments to break resistance mechanisms.

### 5.3 Chromatin remodeling, 3D genome architecture, and transcriptional plasticity

Beyond DNA methylation and covalent histone modifications, another layer of epigenetic regulation involves ATP-dependent chromatin remodeling complexes (Mashtalir et al., 2018). These complexes (e.g., SWI/SNF, ISWI, CHD families) reposition or evict nucleosomes, thereby changing the accessibility of DNA to transcription factors and repair machinery (Barisic et al., 2019; Clapier et al., 2017). In cancer, subunits of chromatin remodelers are frequently mutated–for instance, ARID1A, ARID1B, and PBRM1 (components of the SWI/SNF complex) are mutated in various cancers. In GBM, mutations in classical chromatin remodelers are not as common as in some other tumors, but there are notable exceptions: ATRX (a chromatin remodeler involved in telomere maintenance and deposition of histone H3.3) is mutated in a large fraction of lower-grade gliomas and secondary GBMs with IDH mutation, leading to the ALT (alternative lengthening of telomeres) phenotype (Aguilera and López-Contreras, 2023; Schwartzentruber et al., 2012). However, ATRX mutations are rare in primary GBM. Instead, primary GBMs often have alterations in chromatin regulators indirectly *via* amplification or pathway activation. For example, the PI3K/AKT pathway (often hyperactivated by EGFR mutations or PTEN loss in GBM) can phosphorylate and alter the activity of chromatin modulators like BRG1/BRM (the ATPase subunits of SWI/SNF) (Nirala et al., 2023).

Another aspect is the chromatin state variability within a tumor. Single-cell chromatin accessibility assays (like ATAC-seq) on GBM have revealed that different clones within the same tumor can have distinct accessible regions, correlating with different transcriptional programs (e.g., a clone with open chromatin at MES-related genes vs. another with open chromatin at neuronal lineage genes) (Patel et al., 2014). This accessibility heterogeneity likely underlies the tumor’s ability to adapt to therapy–if radiation kills one subpopulation, another with a different chromatin state can survive and repopulate. It underscores why combination epigenetic therapies might be needed: targeting multiple epigenetic mechanisms at once to prevent the tumor from simply switching strategies.

One interesting emerging area is the role of three-dimensional genome architecture (the physical folding and looping of DNA in the nucleus) in GBM (Xu et al., 2022). This is orchestrated by proteins like CTCF and cohesin which are architectural proteins, and modifications such as H3K27ac mark super-enhancers that drive oncogene expression (Li et al., 2020). GBMs have been found to have unique super-enhancers, for example, around the gene SOX2 (critical for GSCs) or EGFR (Mao et al., 2023; Zhu et al., 2021). Targeting these super-enhancers (perhaps *via* BET bromodomain inhibitors that detach acetylation readers at enhancers) could profoundly reduce expression of these oncogenes. In fact, BET inhibitors like OTX015 (birabresib) and CC-90010 (trotabresib) have been tested in GBM patients (Omuro and DeAngelis, 2013). While OTX015 had limited efficacy (the trial was terminated for lack of efficacy) (Berenguer-Daizé et al., 2016), trotabresib has shown good brain penetration and tolerability, establishing a recommended phase 2 dose in combination with TMZ/RT (Vieito et al., 2022). The hope is that BET inhibition will temper the transcription of multiple growth-promoting genes simultaneously (like c-Myc, which is not mutated in GBM but is often highly expressed due to amplifier enhancers). Indeed, BRD2/3/4 (BET proteins) regulate many pathways in GBM–inhibiting them not only reduces proliferation but also can reduce invasion and even modulate the immune microenvironment (Chen et al., 2024).

Finally, transcriptional plasticity–the ability of GBM cells to reprogram their transcriptome–often relies on permissive chromatin landscapes. When therapy is applied, stress pathways (like p53, AP-1, NF-κB) activate, and if the chromatin is open at certain loci, cells can swiftly upregulate survival genes (Li S. et al., 2021; Pouyan et al., 2025). Chromatin remodelers and histone modifiers are integral to this rapid response. For instance, after radiation, surviving GBM cells have been observed to upregulate genes involved in DNA repair and cytokine signaling; studies show increased H3K27ac at promoters of these genes post-radiation, indicating active enhancer usage (Li X. et al., 2021; Yuan et al., 2023). Using an HDAC inhibitor at the time of radiation may prevent the proper chromatin compaction needed for efficient DNA repair, thereby trapping cells in a more vulnerable state. Alternatively, using a PARP inhibitor (targeting another facet of DNA repair) can synergize with radiation, and there is evidence that PARP1 activity is somewhat contingent on chromatin states–hyperacetylated chromatin might make PARP1 more engaged but also more targetable by inhibitors (Demin et al., 2021).

In conclusion, chromatin remodeling and histone modifications create a layered regulatory system that GBM exploits for malignancy and treatment evasion. Epigenetic heterogeneity ensures that within one tumor, some cells are poised to survive almost any single therapy. Thus, the path forward likely involves multi-modal therapy: hitting the tumor with radiotherapy, plus epigenetic modulators that flatten the tumor’s adaptive landscape, forcing it into a state where it cannot easily switch phenotype or repair damage, leading to more complete tumor cell kill.

## 6 Epigenetic-targeted therapies as radiosensitizers in GBM

Targeting epigenetic regulators in GBM has dual potential: directly inhibiting tumor growth and enhancing the effects of standard therapies like radiation and chemotherapy. Existing studies have shown the effect of the novel heparinase inhibitor RDS 3337 on the balance of apoptosis and autophagy in U87 glioblastoma cells. It indicates that RDS 3337 regulates the interaction between autophagy and apoptosis, revealing a new epigenetic - microenvironment regulatory axis (Manganelli et al., 2023). Given GBM’s intrinsic radioresistance and the role of epigenetics in that resistance, considerable research has focused on combining epigenetic drugs with radiotherapy–a strategy aimed at radiosensitization. Below, we review key classes of epigenetic-targeted therapies and the evidence supporting their use in GBM, particularly highlighting any radiosensitization effects observed in preclinical or clinical studies.

### 6.1 Histone deacetylase (HDAC) inhibitors

HDAC inhibitors (HDACis) were among the first epigenetic drugs tested in GBM and remain one of the most extensively studied classes (Ho et al., 2020). In preclinical GBM models, HDACis have shown the ability to slow tumor cell proliferation, induce differentiation of GSCs, and crucially, to enhance radiation-induced cell killing (Zhao et al., 2020). Mechanistically, HDAC inhibition can diminish the efficiency of DNA double-strand break repair by preventing deacetylation of histones at damage foci (persistent acetylation can impede repair protein recruitment) and by modulating the expression of DNA damage response genes. For example, one study found that vorinostat treatment led to hyperacetylation of histone H3 and correlated with increased markers of DNA damage (γ-H2AX) after irradiation, indicating unrepaired breaks (McClure et al., 2018). Indeed, HDACis are known to cause a phenomenon called “radiation hyper-sensitivity” in cancer cells by trapping them in a vulnerable cell cycle phase and impairing repair–this has been observed in lung cancer models (Solta et al., 2023) and also hinted in GBM.

Multiple ongoing trials are now exploring HDACis with radiation. For instance, entinostat (class I selective) and chidamide (class I HDAC inhibitor used in China) are being tested for their radiosensitizing properties. There is also interest in combining HDAC inhibition with other pathway inhibitors. A preclinical study showed that combining an HDACi with an ATM kinase inhibitor (ATM is key in DNA damage response) dramatically increased radiosensitivity in GBM cells (Cassandri et al., 2024). This concept of dual targeting–one hitting chromatin/DNA repair, another the checkpoint signaling–could overcome redundant resistance mechanisms.

The limitations of HDACis in the past for treating glioblastoma lie in its side effects - it can cause fatigue, thrombocytopenia and gastrointestinal problems, not all HDAC inhibitors have good BBB penetration ability, tumor cells can easily develop acquired resistance through multiple mechanisms, and HDACis are usually not highly selective (Yang et al., 2025). Interestingly, some HDACis (like valproate) may have radio-protective effects on normal brain while radiosensitizing tumor, due to different baseline acetylation states–though this is not definitively proven. In summary, HDAC inhibitors are among the most promising epigenetic radiosensitizers in GBM, with substantial preclinical evidence and emerging clinical data suggesting they can improve outcomes when appropriately combined with radiotherapy (Qiu et al., 2017).

### 6.2 DNA methyltransferase (DNMT) inhibitors

Drugs that inhibit DNA methyltransferases, such as 5-azacytidine (azacitidine) and 5-aza-2′-deoxycytidine (decitabine), are standard treatments in certain leukemias and have been studied in solid tumors including GBM(Christman, 2002; Zhu et al., 2025). These hypomethylating agents get incorporated into DNA (or RNA for azacitidine) and trap DNMT enzymes, leading to passive loss of DNA methylation over successive cell divisions. The rationale in GBM is that DNMT inhibitors could revert the silencing of tumor suppressor genes and possibly counteract the adaptive methylation changes that confer resistance (Lopez-Bertoni et al., 2015).

Preclinical evidence: In GBM cell lines, decitabine has been shown to induce expression of endogenous retroviral elements, thereby stimulating an interferon response that can attract immune cells (a phenomenon exploited in trials combining DNMT inhibitors with immunotherapy) (Lofiego et al., 2024). Decitabine can also upregulate genes that sensitize cells to damage, for example, the TP53 pathway or pro-apoptotic genes. There are not many direct studies of DNMT inhibitors purely as radiosensitizers in GBM, but some clues exist: a general study in cancer cell lines found that pretreatment with DNMT inhibitors enhanced radiosensitivity in a variety of cells by promoting more apoptosis and less cell cycle arrest after radiation (Kang et al., 2019). In glioma models, epigenetic preconditioning with decitabine has been reported to synergize with subsequent TMZ therapy (Gallitto et al., 2020). This suggests a similar approach could be tried with radiation: pre-treat the tumor with a demethylating agent to “loosen up” the epigenome, then apply radiation to inflict damage that the cells are less equipped to repair or survive.

Clinically, DNMT inhibitors have not yet been widely used in GBM, largely due to concerns about crossing the blood–brain barrier and systemic side effects. The mechanism of action of DNMT inhibitors is that after embedding into DNA, they irreversibly bind to and deplete DNMT enzymes, thereby triggering DNA demethylation. However, the effects of this process are indirect and delayed. The drug first needs to be integrated into the replicating DNA, and its effect takes one to two cell cycles to manifest. This has different effects on tumor cell populations with fast or slow cell cycles and cannot achieve rapid killing (Li et al., 2025). However, a novel formulation–guanine-rich oligonucleotides (Zebularine derivatives) or nanoparticle-encapsulated decitabine–might circumvent some issues. One study in abstract form mentioned a decitabine nanoconjugate that could sensitize GBM cells, implying that smarter delivery systems are being developed (Cheray et al., 2025).

In summary, while the concept of using DNMT inhibitors to radiosensitize GBM is appealing, especially to undo methylation-driven resistance, clinical evidence is still sparse. More studies–possibly using next-gen agents or combination with other epigenetic drugs (like simultaneous HDAC and DNMT inhibition to broadly reactivate silenced genes) – are needed. One interesting idea is a “one-two punch”: use a DNMT inhibitor to demethylate and expose cancer-testis antigens or viral elements, then radiotherapy not only kills cells but also releases neoantigens from those now-expressed elements to stimulate immunity against the tumor. This could turn the radiated tumor into its own vaccine, aided by the prior epigenetic reprogramming. Such creative approaches embody the future of epigenetic therapy in GBM.

### 6.3 Bromodomain and extra-terminal (BET) inhibitors

BET inhibitors target the bromodomain family proteins BRD2, BRD3, BRD4 (and BRDT) which read acetylated lysines on histones and recruit transcriptional complexes to promoters and enhancers (Fu et al., 2021). Of these, BRD4 is a key regulator of transcription elongation and super-enhancer function, including driving expression of c-MYC and other growth genes. GBM cells often rely on BRD4 for sustaining high levels of oncogenic transcription programs (Muhar et al., 2018).

Preclinically, BET inhibitors like JQ1 showed that GBM cell proliferation can be curtailed and GSC self-renewal impaired by blocking BRD4. Moreover, BRD2 inhibition has specific effects on invasiveness: as noted earlier, BRD2 *via* NF-κB signaling promotes mesenchymal transition and invasion in GBM (Marchant et al., 2023). A BRD2-selective inhibitor, GSK620, was shown to reduce GBM invasion and importantly to synergize with both temozolomide and radiotherapy, enhancing treatment efficacy (Singh et al., 2023). This suggests that BET inhibitors can act as multi-purpose sensitizers.

The principle behind BET inhibitors as radiosensitizers lies in their transcriptional repression of DNA damage response and survival genes. After radiation, cells typically induce expression of genes to cope with stress; BRD4 is required for maximal induction of many of these. If BRD4 is inhibited, cells may fail to upregulate, say, a key DNA repair enzyme or an antioxidant protein, thereby succumbing to damage (Doroshow et al., 2017). For example, one can imagine that a gene like RAD51 (involved in homologous recombination repair) has a super-enhancer that needs BRD4 – JQ1 could dampen RAD51 upregulation after radiation, leading to more unrepaired DNA breaks and cell death (Shioi et al., 2024; Zou et al., 2022). Additionally, BET inhibitors can modulate the immune microenvironment: GBM is immunosuppressive partly *via* expression of checkpoint ligands and cytokines, some of which are regulated by BRD4 (like IL-6, IL-8). By reducing these, BETi might make the tumor more immunologically “hot” post-radiation, aiding anti-tumor immunity (Smith et al., 2024).

It is worth noting newer compounds like bromodomain degraders (PROTACs) targeting BET proteins are being developed, which might be more effective than classical inhibitors. *Zhang et al.* referenced several such degraders that showed strong anti-GBM activity in models (Zhang et al., 2022). A BET degrader delivered *via* nanoparticles could cross the BBB better and actually remove the protein entirely (Jiang et al., 2025). Although these are early-stage, they illustrate the direction of the field–moving beyond reversible inhibition to induced protein degradation for a more complete shutdown of epigenetic readers.

In conclusion, BET inhibitors remain a promising epigenetic tool against GBM, especially in combination settings. The limitations of BET inhibitors lie in their poor penetration of the BBB, the short half-life of some BET inhibitors in the body, and the need for frequent or high-dose administration to maintain an effective therapeutic concentration, *etc.* The initial clinical setbacks have provided lessons, and ongoing trials with improved compounds and rational combinations (with radiation, chemo, or immunotherapies) will determine if this class can fulfill its potential as a radiosensitizer. There is sound scientific rationale, given their ability to turn off critical pro-survival genes and invasion programs that otherwise undermine therapy.

### 6.4 EZH2 inhibitors and LSD1/PRMT5: key histone methylation targets

Targeting histone methyltransferases in GBM has centered largely on EZH2, as it is the most obviously overexpressed and tied to malignancy. As discussed, EZH2 catalyzes H3K27me3 to silence genes. In GSCs, EZH2 keeps differentiation pathways off and supports radioresistance *via* the MELK-FOXM1-EZH2 axis (Kim et al., 2015; Kim et al., 2024). Thus, inhibiting EZH2 could release the brake on differentiation and make GSCs less able to recover from therapy.

A study by *Kim et al.* showed that genetic knockdown of EZH2 radiosensitized GSCs, and clinically, GBM recurrences after radiation had higher EZH2, supporting that radiation may select for EZH2-high clones (Kim et al., 2015).

Tazemetostat (EPZ-6438) is an FDA-approved EZH2 inhibitor for certain sarcomas and lymphomas; in GBM, a Phase I trial in combination with TMZ was conducted (Gounder et al., 2020). While safe, it did not show significant benefit in unselected GBM. Possibly, only a subset of GBM (e.g., those with high EZH2 or specific methylation patterns) would benefit, pointing towards the need for biomarker selection. An interesting case from a PRMT5 inhibitor trial (PRT811) noted a complete response in a patient with an IDH1-mutant GBM (Xiao et al., 2019) – IDH-mutant tumors often have low EZH2 due to their hypermethylated state, raising the idea that maybe combining EZH2 inhibition with other targeted agents is more useful in IDH-wildtype context. Other histone modifiers of interest are in Table 2.

**TABLE 2:** Epigenetic-targeted drugs and evidence for radiosensitization in glioblastoma.

Drug class	Representative agents	Mechanism of action	CNS penetration	Preclinical evidence	Clinical evidence	Potential spatial niche target
HDAC inhibitors	Vorinostat, Panobinostat, Quisinostat, Valproic acid	Increase histone acetylation → open chromatin, impair DNA repair, enhance apoptosis	Moderate to good	Radiosensitization in GBM cell lines, xenografts; enhanced DDR inhibition	Vorinostat in Phase I/II trials, VPA retrospective evidence with RT	Hypoxic Core, PVN
DNMT inhibitors	Decitabine, 5-azacytidine	Demethylate DNA, reactivate silenced genes, viral mimicry → immune activation	Limited	Preclinical radiosensitization, synergy with TMZ/RT	Early trials in hematologic malignancies; limited GBM data	Hypoxic/edge regions
BET inhibitors	JQ1, OTX015, Trotabresib	Block BRD4 binding at super-enhancers, suppress stress-induced transcription	Good (Trotabresib BBB-penetrant)	Preclinical synergy with RT and PARPi; reduce invasion/mesenchymal shift	Ongoing trials in solid tumors, early CNS safety data	Invasive Edge, immune interface
EZH2 inhibitors	Tazemetostat, GSK126	Block H3K27me3, de-repress differentiation, reduce stemness	Moderate	Radiosensitization of GSCs; synergy with RT	Pediatric HGG/GBM early-phase studies	PVN, GSC-rich areas
Other epigenetic drugs	LSD1 inhibitors, G9a inhibitors, dual HDAC/PI3K inhibitors	Modulate differentiation, apoptosis, immune signaling	Variable	Preclinical GBM models with radiosensitization effects	Mostly in development	Edge + PVN niches

In summarizing this section, epigenetic-targeted therapies offer a versatile toolkit for overcoming GBM resistance. Each class (HDACi, DNMTi, BETi, EZH2i, *etc.*) tackles a different facet of the tumor’s protective machinery. The challenge is to integrate them with standard care in a way that improves efficacy without undue toxicity. The most encouraging data so far come from HDAC inhibitors (multiple trials showing at least safe combinability and hints of improved survival) and from preclinical studies like quisinostat’s that clearly demonstrate radiosensitization. EZH2 inhibitors hold logical appeal for GSC targeting, and BET inhibitors could suppress the adaptive transcriptional responses after irradiation. As new epigenetic drugs (with better brain penetration and specificity) emerge, the prospects for effective radiosensitization in GBM improve. The next stage will involve biomarker-driven trials to identify which patients are most likely to respond to a given epigenetic combo, a topic we will explore in the future directions.

## 7 Future directions

Advancing treatment for GBM will require innovative strategies that integrate knowledge of spatial biology and epigenetic regulation. Below, we outline several future directions that hold promise for overcoming the challenges discussed.

First, spatial multi-omics and precision mapping of heterogeneity to effectively target spatial and epigenetic heterogeneity, we first need to comprehensively map it in individual tumors. Emerging spatial multi-omics technologies enable profiling of gene expression, protein markers, and even epigenetic modifications with spatial resolution. Techniques such as spatial transcriptomics, multiplex immunofluorescence, spatial mass spectrometry, and spatial ATAC-seq can reveal how different regions of a GBM differ molecularly. Recent studies have applied spatial transcriptomics to GBM and revealed multi-layered concentric organization. The goal is that, in the near future, a GBM patient’s resected tumor could be subjected to rapid spatial multi-omic analysis, yielding a “map” of which regions harbor radioresistant cell populations. With such a map, therapy can be precisely tailored–this is the essence of precision oncology for GBM.

Second, targeted delivery and regional therapies. One of the biggest hurdles in GBM treatment is ensuring therapies reach infiltrative tumor cells beyond the resection cavity and across an intact BBB. Future strategies are focusing on targeted delivery systems: nanocarriers, viral vectors, or cell-based delivery that can ferry therapeutic agents to specific tumor regions. For instance, nanoparticles can be functionalized with ligands that bind preferentially to markers of the invasive edge. Hypoxia-activated prodrugs selectively. Emerging technologies such as ultrasound that can open the blood-brain barrier. By applying focused ultrasound to the target brain region, the tight connections of the blood-brain barrier can be physically and temporarily relaxed, thereby increasing its permeability. It might become the core of solving the problem (Gasca-Salas et al., 2021).

Third, adaptive and regional treatment strategies. Given how GBM cells can shift phenotypes under treatment pressure, an emerging concept is adaptive therapy–altering treatments over time or region based on tumor’s response. Instead of treating the tumor as a uniform entity, one strategy is to treat it as multiple sub-tumors each requiring a different approach. For example, one might use deep learning on radiographic images to infer different habitats within the tumor. Each of these could be targeted differently: the enhancing core gets a certain radiosurgery boost plus maybe an HDACi; the non-enhancing infiltrative area gets immunotherapy plus an EZH2 inhibitor, *etc.* This is akin to individualized compartmental therapy.

## 8 Conclusion

Glioblastoma exemplifies the dual challenges of spatial and epigenetic heterogeneity in cancer therapy. Distinct tumor microenvironments–from hypoxic cores to invasive edges and perivascular niches–harbor cellular subpopulations with unique epigenomic profiles and therapy sensitivities. These resilient niches underlie the failure of uniform treatment approaches and drive recurrence. Meanwhile, epigenetic dysregulation (DNA methylation changes, histone modification imbalances, chromatin remodeling defects) endows GBM cells with phenotypic plasticity and adaptive resistance to radiation and chemotherapy. Together, these factors create a moving target for therapy.

In this review, we have detailed how spatial heterogeneity influences GBM’s response to radiotherapy, highlighting that radioresistance is not a monolithic trait but a regional phenomenon sculpted by the tumor microenvironment (e.g., hypoxia-induced radioresistance in the core vs stem-cell-mediated resistance in perivascular zones). We have also summarized the major epigenetic alterations in GBM–such as MGMT promoter methylation (a predictive biomarker for chemoradiotherapy), widespread histone deacetylation and gene silencing contributing to therapy resistance, and the pivotal role of modifiers like EZH2 in maintaining a resistant stem-like state–and linked these to potential interventions. Epigenetic-targeted therapies, particularly HDAC inhibitors, have shown the ability to sensitize GBM cells to radiation by impairing DNA repair and promoting apoptosis. Other agents like BET bromodomain inhibitors can suppress pro-tumor transcriptional programs and may prevent the emergence of resistant phenotypes post-irradiation. EZH2 inhibitors and DNMT inhibitors hold promise in eroding the epigenetic underpinnings of GSC-driven recurrence and immunosuppression, especially when deployed in combination strategies.

In conclusion, overcoming GBM’s lethality will require confronting its heterogeneity head-on. By decoding the spatial and epigenetic code of GBM, we can develop therapies that hit the tumor’s weak points in every niche. Epigenetic therapies add a powerful dimension to this fight–they can reprogram tumor cells from within, strip them of their adaptability, and render them more susceptible to existing treatments. Combined with precise delivery and adaptive planning, there is hope that we can significantly extend survival and maybe achieve long-term control in what is today an incurable cancer. The path is challenging, but the convergence of spatial biology and epigenetic therapy represents a promising frontier in the quest for more effective GBM treatment. As our understanding deepens and new tools emerge, we inch closer to turning GBM into a disease that can be managed and eventually defeated, rather than a swift and inescapable fate.
